# Removal of Foreign Bodies from the Surface of the Eye and Lids

**Published:** 1884-05-20

**Authors:** S. L. Frank

**Affiliations:** Surgeon to the Baltimore Eye, Ear and Throat Charity Hospital, Etc.


### REMOVAL OF FOREIGN BODIES FROM THE SURFACE OF THE EYE AND LIDS.

By S. L. Frank, M. D.,
Surgeon to the Baltimore Eye, Ear and Throat Charity Hospital, Etc.

(*Read before the Clinical Society of Md. Jan. 4, 1884.*)

A few days ago a patient called to see me with the statement that his family physician, whom he had consulted the day before about the pain in his right eye, told him he had a stye coming on the lid, which was the cause of his suffering. Finding no relief from the poulticing ordered, he called to see me.

Upon examination I found no stye but a foreign body (particle of iron) upon the cornea, which I removed, and the trouble was at an end.

This mistaken diagnosis has recalled a number of like nature which have come under my notice the last few years.

This is a state of affairs which I think ought not to exist. A general practitioner should make himself at least perfectly capable of diagnosing a foreign body in these regions, and in many cases be able to remove them. Of course I leave out of the question entirely the removal of foreign bodies from the interior of the eye or those cases where the foreign body, although in the cornea, has also passed partly into the anterior chamber. It is for this reason that I have confined the subject this evening to the *surface* of the eye only. A general practitioner should no more be expected to deal (successfully) with foreign bodies within the eye than he is expected to do resections of bones, ovariotomies, lithotomies or other serious operations.

The discovery of foreign bodies is easy in most of the cases with the naked eye, the patient being seated near the window, in a good light, or at night by oblique illumination. When very minute, they can be seen with a magnifying glass, and the removal in most of the cases is easily effected.

Why is it, then, that these mistakes are so often made of not recognizing the cause of the trouble ? Is it a dread of touching the eye or ignorance how to look for foreign bodies ? The latter in some cases that I have seen was the more likely. Thus I recall a case where a mould, in which red-hot iron had been poured, exploded and some particles of the hot iron flew into the inner canthus of the eye and lodged on the conjunctiva and remained there for four days; although fully the size of a small tack-head, two physicians, whom the man had consulted, without examining the eye, ordered an astringent wash. The removal of the piece of iron by me, with a little sweet oil instilled afterwards, ended the trouble. This picture is not overdrawn. I am sure my colleagues present this evening can recall also many cases in which patients with foreign bodies on cornea were treated for eye diseases which did not exist.

Now, where do we generally find foreign bodies and consequently where must we look for them ? As a rule on the cornea or under the upper lid. If the patient is a mechanic he probably will tell you that a piece of iron or steel has flown into his eye, either whilst at work or grinding his tools. In this case it will be an easy matter to seek for the foreign body.

Where are we to look first ? As a rule at the cornea, that being the spot where the body most frequently lodges.

The patient being seated facing the window, separate the lids and carefully examine the cornea, letting the eye follow a finger in all directions so as to throw the light well on all its parts; if not to be found in this way with the naked eye then examine by oblique illumination, a lens of two or three inches focus being used to concentrate the light upon the different parts of the cornea; if the foreign body is so minute as not to be seen even in this way, it can be still more magnified by concentrating the light, as before stated, through a three-inch lens and at the same time examining the cornea by looking through a 2 or 2½ inch glass. In this way it will be impossible to overlook a body, no matter how small. Some of the smallest I have ever seen and a number of which could not otherwise be detected, have been tiny pieces of percussion caps fired from toy or other pistols.

Dr. Noyes, in his work on Diseases of the Eye, pages 213 and 214, gives the drawing of a contrivance with a lens attached to a ring which can be put on the index finger of the left hand and thereby the light concentrated upon the cornea whilst holding the lids apart with the fingers, so as to bring out the spot where the foreign body is lodged and assist in its removal.

Should the patient be a traveler, coming by rail the probabilities are we are dealing with cinders from a locomotive, and it is surprising what an amount of irritation and inflammation is sometimes seen in these cases; here we very often find the foreign body under the upper lid near the edge. Having struck the eye it is usually carried by the movements of the ball under the upper lid where it remains and keeps up a continual friction upon the eye.

I know of no simple trouble of the eye where such profuse thanks are as often given by the patient as in the removal of cinders, the relief being of course almost instantaneous.

How are we to get at the corpus delicti when under the upper lid? The simplest method, and I think the least disagreeable, is to hold a probe or pen-handle or any hard substance horizontally across the middle of the upper lid with the right hand (in the lower lid it is of course not necessary as it is easily pulled down and outwards); then by pressing the probe backwards and downwards and catching the lashes or edge of upper lid and telling the patient to look down at the same time, pull the lashes and edge of lid upwards with the thumb and index finger of left hand; by this manœuvre the lid is easily turned over. We have now no trouble in examining the lid to the retrotarsal fold; as a rule the cinder or whatever else it may be, is found lying just beyond the edge of the lid on the conjunctival surface, and can easily be wiped away either with a bit of cotton wrapped around a piece of wood or end of handkerchief.

Should the patient be neither a mechanic nor traveler, then the history of the case, and suddenness of the discomfort, will attract our attention to the possibility of a foreign body on the eye; in fact, it is a rule in all cases to examine cornea, conjunctiva and lids, before going to the interior.

Having found the foreign body on the cornea, what instrument do we need and how shall we remove it? If it is a bit of cinder, metal or other substance merely on the surface of the cornea, it can be easily removed by a simple method which was first brought to my attention by my friend, Dr. Russell Murdoch, and consists of a wooden tooth-pick or match around one end of which a little absorbent cotton is wrapped; this being a soft substance, it pains but very little, if at all, when carried across the cornea and the foreign body is removed.

Should the foreign body be imbedded in the substance of the cornea, it can be removed by a spud, cataract needle or a scoop, with which it is easily picked out of its bed; the latter is my favorite instrument, especially where the metal was hot when it struck the eye, and has burnt the tissue around it; the latter can be thoroughly cleaned out with this instrument. My method of placing patients is generally seated, whilst I stand behind them and let the head rest backwards against my chest (of course facing the light and near a window), then separate the lids with the fingers of the left hand and at the same time with middle finger press upon the eyeball sufficiently to steady it; in this way no assistant will be necessary. With a child or very nervous person it may be necessary to æsthetize them.

If the above procedure cannot be carried out, holding lids and eyeball steadily, a lid-holder can be introduced and the eyeball steadied with forceps. Of course this method is more painful than the other, but is perhaps the one a beginner or a person less experienced would prefer.

After removing particles of iron, a rust stain sometimes remains; this need not be removed, as it will disappear of itself in a few days.

If a good deal of epithelium has been scraped off in getting out the foreign body, laying some of the corneal nerves bare, or the eye is very sensitive, it is well to put a drop of atropine in the eye and then bandage it for a day or so. This will be all that is necessary in most cases.

If the foreign body is on the ocular conjunctiva it can easily be removed, if not too firmly imbedded, with the spud, cataract needle or bit of absorbent cotton.

In the October number of the Centralblatt der Augenheilkunde, this year, Hirschberg, in a review of Simeon Snell's work on the electro-magnet and its employment in ophthalmic surgery, adds a report of some additional cases of his own; these all refer to the foreign body in the interior of the eye. At the same time he reports two cases (Nos. 18 and 19), in the former of which the patient's physician had made twenty-five ineffectual attempts to remove the triangular piece of iron imbedded deeply in the parenchyma of the cornea and part of it in the anterior chamber also. Hirschberg inserted a lid-holder, and holding the eye with forceps he shaved off part of the cornea above the foreign body with the keralom and then extracted the foreign body with the electro-magnet, the piece weighing three milligrams. Case No. 19 was a similar case and was relieved in the same manner, the foreign body weighing 1-5 milligram. The first case left the hospital in twenty-four hours, the eye being free from irritation. The second case had slight irritation of the eye for a week from rust, which had remained behind.

I merely mention these cases in order to bring before you this new method of removing iron from cornea, the most recent triumph in modern ophthalmology.

